# New digital tools for assessing neuropsychological executive functioning in old and new addictions. an exploratory study

**DOI:** 10.1192/j.eurpsy.2021.1946

**Published:** 2021-08-13

**Authors:** M. Balconi, M. Sansone, L. Angioletti

**Affiliations:** International Research Center For Applied Cognitive Neuroscience (irccan), Research Unit In Affective And Social Neuroscience, Department Of Psychology, Catholic University of the Sacred Heart, Milano, Italy

**Keywords:** Executive functions, neuropsychology, digital assessment, Addiction

## Abstract

**Introduction:**

Nowadays new tools suitable for exploring executive functioning (EF) of behavioral addicted individuals are needed.

**Objectives:**

This study tests a novel digital assessment battery that can be easily and remotely adopted by neuropsychologists working in the field of addiction.

**Methods:**

Twenty-three participants were divided into two groups, balanced for age and education: an experimental (EXP) group of 13 patients with gambling behavior, and a control (CNT) group of 10 healthy subjects. A neuropsychological battery including 5 neuropsychological tests (measuring long- and short-term verbal memory, working memory, cognitive flexibility, verbal and non-verbal fluency, attention), and a behavioral task (modified Go/NoGo task with addiction-related stimuli) was digitally administered. Anxiety, depression, and impulsivity levels were collected before the evaluation.

**Results:**

Significantly higher scores were found for repetition errors in the short-term verbal memory test, in the EXP subjects compared to controls. Higher reaction times were found in the Go/No-Go task for the EXP compared to CNT, with significant differences for neutral and addiction-related (cocaine, THC) stimuli. Furthermore, EXP showed higher impulsivity scores.

**Conclusions:**

Although the study was only exploratory, the significant results could support the validity of this new digital tool. Besides, we could conclude that memory impairment and attentional bias in inhibitory control tasks could cover a significant role in new and old addiction and that impulsivity could represent a critical factor in explaining the relationship between EF impairment and addiction. Lastly, this study contributes not only to the understanding of EF impairment in addictions but also in the delivery of remote suitable digital neuropsychological testing.

**Disclosure:**

No significant relationships.

